# Targeting WDR5: A WINning Anti-Cancer Strategy?

**DOI:** 10.1177/2516865719865282

**Published:** 2019-07-18

**Authors:** Erin R Aho, April M Weissmiller, Stephen W Fesik, William P Tansey

**Affiliations:** 1 Department of Cell and Developmental Biology, School of Medicine, Vanderbilt University, Nashville, TN, USA; 2 Department of Biochemistry, School of Medicine, Vanderbilt University, Nashville, TN, USA

**Keywords:** Cancer therapy, chromatin, epigenetic mechanisms, histone methylation, MYC, small molecule inhibitors, WDR5

## Abstract

WDR5 is a component of multiple epigenetic regulatory complexes, including the mixed lineage leukemia (MLL)/SET complexes that deposit histone H3 lysine 4 methylation. Inhibitors of an arginine-binding cavity in WDR5, known as the WDR5-interaction (WIN) site, have been proposed to selectively kill MLL-rearranged malignancies via an epigenetic mechanism. We discovered potent WIN site inhibitors and found that they kill MLL cancer cells not through changes in histone methylation, but by displacing WDR5 from chromatin at protein synthesis genes, choking the translational capacity of these cells, and inducing death via a nucleolar stress response. The mechanism of action of WIN site inhibitors reveals new aspects of WDR5 function and forecasts broad therapeutic utility as anti-cancer agents.

**Comment on:** Aho ER, Wang J, Gogliotti RD, et al. Displacement of WDR5 from chromatin by a WIN site inhibitor with picomolar affinity. *Cell Rep.* 2019;26:2916.e13-2928.e13. doi:10.1016/j.celrep.2019.02.047. PubMed PMID: 30865883. PubMed Central PMCID: PMC6448596. https://www.ncbi.nlm.nih.gov/pmc/articles/PMC6448596/

## Introduction

Growing awareness of the importance of epigenetic processes in cancer has fueled interest in the idea that targeting the proteins that read, write, and erase epigenetic marks could have therapeutic benefit against a range of malignancies. Two classes of epigenetic drugs have already been approved by the US Food and Drug Administration (FDA) for cancer therapy, more have progressed to late-stage clinical trials, and even more are in preclinical development.^1^ The idea that epigenetic proteins are druggable targets has rapidly moved from concept to reality, and it is not unreasonable to suggest that we are approaching a time where cancer epigenomes can be selectively reprogrammed to reverse the tumorigenic state.

Given the battle that any new drug faces making in it to the clinic, reaching an era of true epigenomic cancer therapy will require a steady stream of new targets, coupled with creative approaches toward their pharmacological inhibition. The last 20 years has seen an explosion in our knowledge of epigenetic players and their mechanisms, and there is no shortage of targets that, on face value, could serve as effective focal points for drug discovery programs. However, this does not mean that epigenetic therapies are as simple as picking targets, finding ways to drug them, and then watch as predictable changes in the malignant epigenome unfold. Even for FDA-approved drugs like DNA methylation inhibitors, for example, it is hard to find compelling evidence that clinical efficacy is directly tied to alterations in DNA methylation status.^2^ For epigenetic processes that are less well understood than DNA methylation, the degree of difficulty in anticipating exactly what a specific inhibitor will do, or what it can be used to treat, is much higher. If, however, a good target is picked and good inhibitors become available, the process of drug discovery itself can hone understanding of epigenetic players and vulnerabilities considerably. Case in point: WDR5.

We recently described a set of potent small molecule inhibitors against the WDR5-interaction (WIN) site of WDR5.^3^ These inhibitors were discovered with the intent of driving changes in histone H3 methylation that would cause certain leukemia cells to differentiate or die. As that project unfolded, however, and potent chemical probes against the WIN site became available, we learned that their mechanism of action was very different from what we expected. Here, we describe how WIN site inhibitors act in leukemia cells, reflect on what this tells us about the functions of WDR5, and speculate on what the utility of this new class of inhibitors may be.

## The Premise: WIN Site Inhibitors for Treatment of MLL-Fusion Cancers

WDR5 is a highly conserved WD40-repeat containing protein that has multiple functions in the nucleus, including scaffolding the assembly of chromatin regulatory complexes linked to gene activation.^4^ Its best-studied role is within the mixed lineage leukemia (MLL)/SET histone methyltransferases (HMT), multi-subunit enzymes that lay down histone H3 lysine 4 (H3 K4) di- and tri-methylation (me2 and me3) at transcriptionally active sites in the genome. The MLL/SET complexes contain a common module made up of WDR5, RBBP5, ASH2 L, and DPY30, usually referred to as “WRAD,” that assembles with one of six catalytic MLL/SET proteins (MLL1–MLL4, SETd1A, and SETd1B). All six MLL/SET family members depend on association with WRAD for full enzymatic activity, and all six carry a conserved WIN motif consensus (“ARA”) that engages a deep arginine-binding pocket on WDR5 called the WIN site.^5^ Interestingly, although WIN site binding is a common feature of MLL/SET proteins, MLL1 preferentially depends on this interaction for its enzymatic activity.^6,7^ It is this preference, together with the potential druggability of the WIN cavity, that first gave rise to the notion that WIN site inhibitors could be discovered that would block MLL1-mediated HMT activity.

Early on, the obvious center stage for WIN site inhibitors was MLL, so-called because leukemic blasts from these patients express cell surface markers associated with both the lymphoblastic and myeloid lineages. MLL is predominantly a pediatric malignancy, and children diagnosed with MLL have dismal outcomes, creating an urgent need for new therapeutic options. MLL cancers are typified by translocations involving chromosome 11q23, which result in fusion of the *MLL1* locus to any one of ~70 different partner genes.^8^ For common translocations, this fusion creates a hybrid transcription factor that drives malignancy, in part, by reactivating expression of developmentally important genes such as the *HOX* loci. Importantly, although hybrid MLL1 proteins lose HMT activity, the untranslocated *MLL1* allele is usually retained in MLL patients, leading to the idea that wild-type MLL1 is essential for tumorigenicity in this context. Logically, if MLL1 is required for leukemogenesis in MLL-fusion cancers, and if its function depends on binding the WIN site of WDR5, then WIN site blockade could be used to specifically inhibit MLL cell viability.

In 2014, Cao et al^9^ became the first to reduce this idea to practice when they described MM-401, a cell-permeable peptidomimetic that binds the WDR5 WIN site with high affinity (K_d_ ~1 nM). As a tool compound, MM-401 comports with all expectations. It selectively inhibits MLL1-mediated HMT activity *in vitro*. It preferentially blocks proliferation of MLL-rearranged leukemia cells in culture. And it induces the expected decreases in H3K4 methylation at the *HOX* genes—decreases that ultimately starve MLL-fusion cells of the vital *HOX* gene products they need to maintain the malignant and stem-like state. The ability of MM-401 to selectively block MLL cancer cell survival, and its compliance with all of the anticipated mechanisms of action, forecast a promising future for drug-like WIN site inhibitors in the treatment of MLL-fusion cancers.

## The Surprise: Predicted Cell Killing by an Unpredicted Mechanism

We used fragment-based screening and structure-based design to discover small molecules that bind tightly and selectively to the WIN site of WDR5.^3,10^ Two of these molecules, C3 (K_d_ ~1 nM) and C6 (K_d_ ~100 pM) were used as chemical probes to explore the biology of WDR5 and the therapeutic potential of WIN site blockade. In *in vitro* HMT assays, C3 and C6 are potent inhibitors of MLL1-mediated HMT activity (the IC_50_ for C6 in this case is ~20 nM), and are at least 250 times more effective at inhibiting MLL1 than any of the other MLL/SET family affiliates. Moreover, when paneled against more than a dozen human and mouse cell lines, both compounds show preferential inhibition of leukemia cells bearing the most common MLL-fusions, MLL–AF4 and MLL–AF9. The biochemical and cell-based selectively of these WIN site inhibitors is thus entirely as predicted. The mechanism of action of these compounds in MLL-fusion cells, on the other hand, is not.

It is worth emphasizing that our approach to mechanism of action studies was different to that taken with MM-401. One important distinction was our assumption that targets for WIN site inhibitors would be genes that are bound by WDR5, and not necessarily those we expect to be regulated by vestigial MLL1 complexes. This distinction turned out to be important, as work from Patricia Ernst’s group subsequently demonstrated that, contrary to expectation, wild-type *MLL1* is not required for survival of MLL-fusion leukemia cells.^11^ A second distinction was that we cast aside the assumption that epigenetic inhibitors necessarily act over a long time frame, and can require multiple rounds of cell division to erase or dilute relevant marks on DNA or histones.^12^ Performing mechanism of action studies days after compound treatment, as done for MM-401, makes it impossible to tease apart cause from effect, and is problematic for modifications like H3K4 methylation, which can change as a result of changes in transcription, direct or indirect. Instead, we measured changes in chromatin-centric processes within minutes and hours of inhibitor treatment, allowing us to monitor primary responses and watch as these unfold to induce cell death.

**Figure 1.**
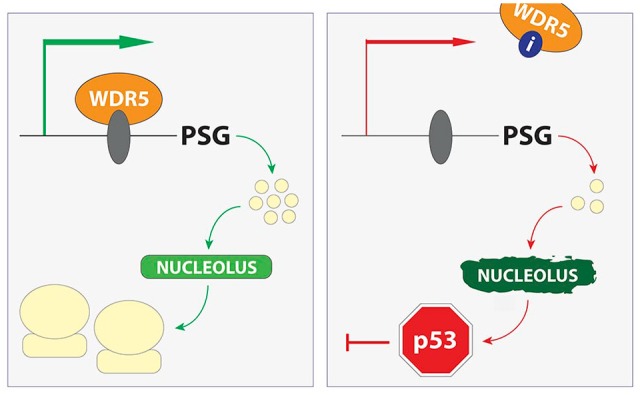
Model for action of WIN site inhibitors. Left panel: WDR5 is bound to chromatin, downstream of the transcription start site (green arrow), at a conserved group of protein synthesis genes (PSG). WDR5 associates with these genes by engaging a chromatin-resident protein (gray oval) via the WIN site. Association of WDR5 with these genes ensures balanced production of ribosome subunits and other nucleolar and translation factors necessary to support appropriate levels of ribosome production. Right panel: WIN site inhibitors (“i”) displace WDR5 from chromatin, reducing PSG expression. This in turn induces nucleolar stress, activating p53-dependent cellular inhibition.

We mapped WDR5 localization on chromatin in MV4:11 cells (MLL–AF4), and were surprised to see that it is not bound to any of the classic developmental loci, including *HOX* genes. Instead, we found that WDR5 associates with a small (~150) but polarized group of genes connected to protein synthesis. This cohort of protein synthesis genes (PSGs) includes about half of the ribosomal protein genes (RPGs), as well as those encoding nucleolar RNAs and translation initiation factors. We saw that WIN site inhibitors rapidly and comprehensively displace WDR5 from chromatin, resulting in changes in the transcription of a subset of WDR5-bound PSGs that can be detected as early as 15 minutes after inhibitor treatment—changes that are not associated with a decrease in histone H3K4 methylation. Consistent with diminished PSG expression, MV4:11 cells display decreased translational capacity within a day of C6 treatment, and this is associated with activation of a nucleolar stress response; a failsafe mechanism that induces p53-dependent cell killing when the supply of ribosome subunits cannot meet the cellular demand. Accordingly, WIN site inhibitors activate p53 in MLL-fusion cells, and the response of these cells to C3 and C6 is stimulated by presence of an intact p53 pathway. Thus, despite our best expectations and the empirical sensitivity of MLL-fusion cells to WIN site blockade, the action of these inhibitors occurs through rapid and persistent decreases in PSG transcription (Figure 1), and seems to have little to do with MLL1, *HOX* genes, or even H3K4 methylation.

## The Insight: What WIN Site Inhibitors Tell Us About WDR5

Before elaborating on the clinical implications of our work, it is worth taking some time to discuss what our study tells us about WDR5 itself. The most obvious new information we learned is that stable association of WDR5 with chromatin is mediated by the WIN site. There are many ways this could occur, the simplest of which would be if WDR5 binds to a WIN motif in a chromatin-resident protein (Figure 1). WDR5 has a very discrete pattern of localization on chromatin, with narrow ChIP-Seq peaks that suggest focal recruitment to specific locations at target loci.^3,13^ Within PSGs, the majority of these peaks occur just downstream of the transcription start site, and they are highly conserved in disparate cell types,^3^ suggesting that both the binding of WDR5 to these genes, and its specific placement within them, is a precise and stably-determined event. Given the pattern of binding, it is entirely possible that placement of WDR5 on chromatin is driven by a sequence-specific DNA binding protein. None of the dozen or so known WIN site binders^4^ have been reported to recruit WDR5 to chromatin, and none of them share this explicit pattern of chromatin binding. If there is a WIN-carrying protein that recruits WDR5 to chromatin, however, it is awaiting discovery, and it is going to have to be discovered the hard way, as there are many thousands of proteins encoded in the human genome that carry an ARA (or similar) sequence. Future experiments to define the full repertoire of WIN site binders, coupled with a detailed exploration of the role of DNA elements in positioning WDR5 at PSGs, are needed to identify the missing piece of this puzzle.

Another conspicuous finding from this work is the connection between WDR5 and PSGs, which appears to be a conserved aspect of the chromatin-binding profile of WDR5. Given its importance in development^4^—and its frequent overexpression in cancer—the ability of a protein like WDR5 to couple epigenetic regulation to enhanced biomass accumulation could have real functional significance, allowing new patterns of gene activity to be met, in lock-step, with commensurate changes in protein synthesis capacity. However, delving deeper into the connection, this simple idea starts to get more complicated. Of the 80 RPGs, only ~40 are WDR5 targets.^3^ If the overt biological role of WDR5 is to enhance protein synthesis capacity by coordinating ribosome protein gene expression, it is unlikely to do so by stimulating production of half of the ribosomal proteins. So what does the biased control of a subset of RPGs by WDR5 mean?

We suggest that the significance of this phenomenon lies not in coordination of ribosomal protein expression, but in the imbalance of ribosome protein subunits it could promote in response to oncogenic threat. We have previously reported that the oncoprotein transcription factor MYC is recruited to chromatin through its interaction with WDR5 (which is mediated via a surface of WDR5 distinct from the WIN site).^13^ MYC promotes transcription of almost all ribosome subunits, as well as many ribosome biogenesis factors.^14^ As MYC levels rise in a nascent cancer cell, the avidity provided by WDR5 could cause a preferential activation of WDR5-bound RPGs, resulting in an excess of these subunits. Alternatively, if binding of WDR5 to these RPGs is a signal-responsive event, and that signal is not received at the time that MYC is induced, this could result in selective activation of the non-WDR5-bound RPGs. In either case, the net effect would be an imbalance in the stoichiometry of ribosome protein subunits entering the nucleolus and induction of p53 via the nucleolar stress response. Given the high rate of flux through the ribosome assembly pathway, such a process could serve as a sensitive counting mechanism for MYC protein levels, and an efficient nuclear trigger to activate p53 at the first signs of aberrant MYC expression.

## The Promise: Clinical Utility of WIN Site Inhibitors

A strong empirical case can be made that MLL-rearranged leukemia cells are sensitive to WIN site blockade,^3,9^ and every indication is that WIN site inhibitors could be developed into effective treatments for MLL cancers. But the precise molecular underpinnings of this sensitivity remain unknown. The mechanism of action we describe for WIN site inhibitors is distinct from all expectations, and there is no direct point of intersection between MLL-fusion oncoproteins and transcriptional processes controlled by WDR5. On one hand, this is an unsatisfying realization, given the motivation behind discovery of these inhibitors. On the other hand, disconnecting the mechanism of action of WIN site inhibitors from MLL-fusion oncoprotein opens a world of potential clinical use for WIN site inhibitors that was not previously imaginable.

The most general prospective utility for WIN site inhibitors relates to their ability to inhibit PSG expression, as enhanced protein synthesis is a prominent recurring feature of cancer. As far back as the 19th century, it was reported that nucleoli are often engorged in cancer cells, which we now recognize to be a result of ribosome biogenesis hyperactivity. We now also know that oncoproteins such as MYC and RAS—and MLL-fusions—drive accelerated biomass accumulation as part of their oncogenic programs, and we appreciate that aneuploidy, together with relentless cell division, create an enhanced demand for protein synthesis in malignant cells that likely pushes proteostatic checkpoints to the brink of collapse. As a result, there is excitement in developing new drugs that impair protein synthesis, and good reason to believe that nucleolar-targeted therapies will be powerful anti-cancer approaches.^15^ WIN site inhibitors, which ultimately restrict the supply or alter the balance of ribosome protein subunits, can be thought of as a type of nucleolar-targeted therapy, and should be expected to have similar or at least overlapping anti-tumor activities. Importantly, however, because WIN site inhibitors act through a fundamentally different mechanism than other nucleolar-targeted drugs, we also expect them to have different properties that could be clinically beneficial, including different on-target toxicities, different mechanisms of resistance, and different patterns of drug synergy. Having a new way to choke translation in malignant cells is thus an important addition to this arsenal of anti-cancer agents.

The mechanism of cell killing we report with WIN site inhibitors in MLL-fusion leukemia cells is largely p53 dependent.^3^ Half of all cancers retain wild-type p53, and having a new way to reactivate or induce p53 in cancer cells can be viewed as a therapeutic positive. Of course, half of all cancers also lack wild-type p53, raising the question of whether WIN site blockade will be ineffective in this context. Here, thinking about WIN site inhibitors as nucleolar targeted therapies is again useful. The overwhelming precedent from other therapeutic approaches targeting protein synthesis is that cancer cells have both p53-dependent and -independent responses to nucleolar stress, and exactly how they respond to nucleolar stress is determined by the specific pathways that remain intact in the cell. Clearly, we have a lot to learn about the mechanisms through which decreased PSG expression can trigger cell death in different contexts, but the beaten path laid out by other nucleolar-targeted therapies gives confidence that WIN site inhibition can be applied to treat cancers with wild-type, mutant, or even missing p53.

In addition, we posit that WIN site inhibitors will also be effective against MYC driven tumors. WDR5 recruits MYC to chromatin,^13^ and it’s not a stretch to predict that if WIN site inhibitors displace WDR5 from target genes, MYC will be displaced in the process. Although our recent work demonstrated that the number of target genes controlled by WDR5 in MV4:11 cells is rather small,^3^ their biological clustering—together with the importance of enhanced protein synthesis as part of the core tumorigenic program of MYC—forecast that WIN site inhibitors should be able to effectively block this crucial aspect of MYC function. One third of all cancer deaths are due to inappropriate MYC expression or activity, and if WIN site inhibitors can be applied in this context, their impact could be profound.

Finally, it is worth emphasizing that, despite everything we learned, and all the surprises along the way, WIN site compounds are potent and selective inhibitors of MLL1-mediated HMT activity. They may also inhibit other enzymatic complexes that involve WDR5, such as the NSL histone acetyl-transferase.^4^ If cancers exist that uniquely depend on the histone marks laid down by MLL1- or NSL-containing complexes, this decidedly non-epigenetic tale of WIN site inhibition may take a profoundly epigenetic turn.
